# Comparison of Lower-Limb Muscle Synergies Between Young and Old People During Cycling Based on Electromyography Sensors—A Preliminary Cross-Sectional Study

**DOI:** 10.3390/s24206755

**Published:** 2024-10-21

**Authors:** Li Kong, Kun Yang, Haojie Li, Xie Wu, Qiang Zhang

**Affiliations:** 1 Department of Rehabilitation, The Affiliated Hospital of Xuzhou Medical University, Xuzhou 221004, China; kl19120207@163.com; 2 Key Laboratory of Exercise and Health Sciences of Ministry of Education, Shanghai University of Sport, Shanghai 200438, Chinawuxie_sus@163.com (X.W.); 3 Institute for Biomechanics, ETH Zürich, 8092 Zürich, Switzerland

**Keywords:** aging, cycling, muscle synergy, non-negative matrix factorization, EMG

## Abstract

The purpose of this study was to analyze the lower-limb muscle synergies of young and older adults during stationary cycling across various mechanical conditions to reveal adaptive strategies employed by the elderly to address various common pedaling tasks and function degradation. By comparing lower-limb muscle synergies during stationary cycling between young and old people, this study examined changes in muscle synergy patterns during exercise in older individuals. This is crucial for understanding neuromuscular degeneration and changes in movement patterns in older individuals. Sixteen young and sixteen older experienced cyclists were recruited to perform stationary cycling tasks at two levels of power (60 and 100 W) and three cadences (40, 60, and 90 rpm) in random order. The lower-limb muscle synergies and their inter- and intra-individual variability were analyzed. Three synergies were extracted in this study under all riding conditions in both groups while satisfying overall variance accounted for (VAF) > 85% and muscle VAF > 75%. The older adults exhibited lower variability in synergy vector two and a higher trend in the variability of activation coefficient three, as determined by calculating the variance ratio. Further analyses of muscle synergy structures revealed increased weighting in major contribution muscles, the forward-shifting peak activation in synergy one, and lower peak magnitude in synergy three among older adults. To produce the same cycling power and cadence as younger individuals, older adults make adaptive adjustments in muscle control—increased weighting in major contribution muscles, greater consistency in the use of primary force-producing synergies, and earlier peak activation of subsequent synergy.

## 1. Introduction

As part of aging, older adults often experience significant functional impairments and difficulties in motor control, primarily manifested as declines in muscle work efficiency, strength, and coordination [1,2,3]. The reduction in muscle mass in older adults not only affects their athletic performance but also increases the risk of falls and injuries [4].

Cycling, a popular form of physical exercise, is highly recommended for older adults [5]. It has proven effective in enhancing muscle power, endurance, and functional ability within this population [6,7]. Nonetheless, challenges such as diminished musculoskeletal capacity, joint limitations, and reduced endurance that older adults face may hinder their participation in high-intensity or prolonged cycling training [8]. Therefore, further research on cycling strategies for older adults can help them better perform power cycling training to improve their functional abilities.

Previous studies have analyzed muscle activity during cycling using electromyography (EMG), focusing on EMG magnitude, activation duration, co-activation duration, and so on to understand muscle activation in older adults. Studies have shown that aging leads to increased co-activation of knee and ankle muscles during cycling [9,10], changed muscle activation amplitude and time [11], and a larger decrease in maximal voluntary contraction after fatigue [12]. Additionally, compared to ankle dorsiflexors, knee extensors exhibit more pronounced age-related decline [13].

However, given that cycling is a closed-chain exercise with a relatively fixed trajectory, simple time–frequency domain analyses may not fully capture subtle changes in muscle activity among older adults. Our study seeks to delve deeper into the synergies and coordination between different muscle groups during cycling, aiming to understand how these interactions contribute to overall performance and how they differ between younger and older adults. This comprehensive analysis may provide valuable insights into the relationship between neuromuscular degeneration and altered exercise strategies with age, helping older adults to better perform power cycling training.

The concept of muscle synergy has been used to solve neuromuscular control problems in locomotion [14,15], with Bernstein defining muscle synergy patterns as large groups of muscles ‘working together’ [16], a definition that has been applied to neural control research through the later development of related linear decomposition algorithms, in particular non-negative matrix decomposition algorithms. The muscle synergy model mathematically reduces a multidimensional signal to a linear combination of several time-invariant muscle synergy vectors and time-varying activation coefficients, and this organization of muscles into such groups has been suggested to help reduce the number of variables manipulated by the brain and reduce the burden on the nervous system [17].

In recent years, muscle synergy has been studied extensively in the elderly population. Previous studies have shown that although aging does not directly reduce the number of muscle synergies, it significantly affects the muscle synergy structure and their variability [18,19,20,21]. For example, in gait analyses, aging led to an increase in the step-to-step and inter-subject variability of activation coefficients [18,22]. Jeon et al. [23] found that ankle joint muscle activation was dominant in older adults, while younger individuals exhibited more prominent hip joint muscle activation. Similarly, research on stair climbing had shown that although the number of muscle synergies remains stable in older adults, the structure and complexity of the synergies change [24]. These changes suggest that although older adults still rely on similar muscle synergy strategies during exercise, the functional properties and control accuracy of these strategies are affected by aging.

Currently, there are relatively few studies on muscle synergy in cycling in older adults. Cycling is a form of aerobic exercise that requires prolonged muscle coordination, with low impact and high functional requirements. Unlike walking, climbing stairs, and sit-to-stand, cycling involves complex movement patterns and a greater range of joint motion, which may make the effects of aging on muscle synergy more pronounced. Previous studies have focused on muscle synergy in static or simple dynamic movement patterns, but the specific effects of cycling, as a form of high-intensity, long-duration exercise, on muscle synergy in older adults have not been fully explored.

This study aims to fill this knowledge gap by applying a non-negative matrix decomposition algorithm to compare lower-limb muscle synergies between young and older adults during cycling. By systematically analyzing muscle synergy, we expect to reveal the specific effects of aging on muscle synergy patterns in cycling. This will not only help us understand how aging alters motor control strategies but may also provide a scientific basis for the development of cycling training and rehabilitation strategies for the elderly population. Given the potential importance of cycling in the health promotion of older adults, the results of this study will provide a valuable reference for optimizing exercise programs and improving exercise capacity in older adults.

Hence, the study was designed to investigate the muscle synergy in young and older adults under various cycling conditions. We hypothesized that (1) there would be the same number of synergies during cycling in both groups and (2) there would be differences in the variability of the synergy activation coefficient of cycling between the two groups.

## 2. Materials and Methods

### 2.1. Participants

Sixteen young (21–25 years) and sixteen older (61–66 years) experienced cyclists were recruited in this study, and their anthropometric data are provided in Table 1. The following inclusion criteria were applied: (1) having more than three years of cycling experience and riding for more than 30 min per day, (2) no lower-limb injury within six months before the experiment, and (3) not engaged in strenuous exercise within 72 h prior to the experiment. This study complied with the Declaration of Helsinki and was approved by the ethics committee of Shanghai University of Sport (No. 102772020RT076). All subjects were clearly informed about the study’s purpose, protocol, and safety measures, and they then signed informed consent forms.

### 2.2. Protocol of Cycling Test

**Figure 1:**
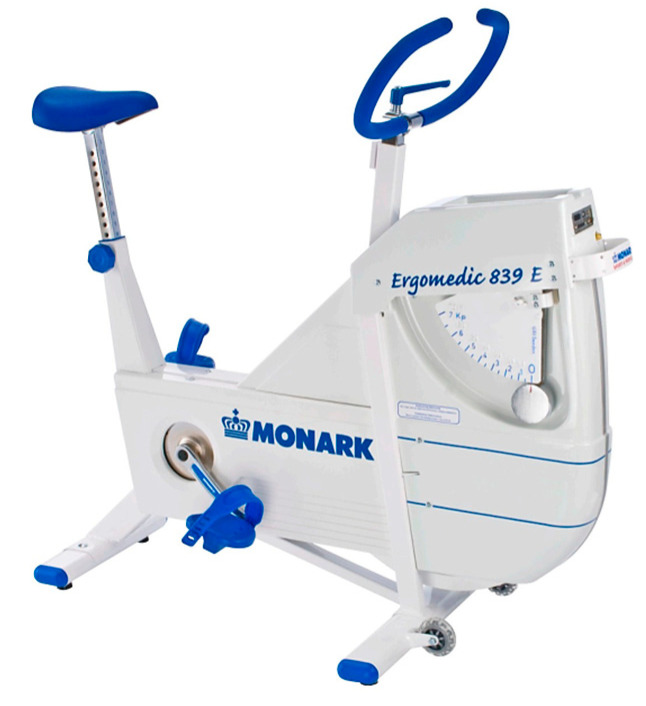
Monark cycle-ergometer.

The subjects wore athletic clothing and sports shoes during the test. All riding tests were carried out using an electromechanically braked cycle-ergometer (MONARK, Monark 839E, Varbergs, Sweden) (Figure 1). In this experiment, we adopted a manually set constant power riding mode, which is able to regulate the resistance according to the change in pedaling cadence. The saddle and handlebars were adjusted according to each participant’s greater trochanter height [25]. Subjects first warmed up by riding at 50 W for 5 min and then rested for 5 min. They were then instructed to perform a 3 min cycling test under each of six different riding conditions: two levels of power (60 and 100 W) and three cadences (40, 60, and 90 rpm). In addition, the order of these six tasks for each subject was randomized by a computer. In each riding condition, subjects were required to reach the target cadence as soon as possible and then maintain it (target cadence ± 5 rpm). These two levels of power accounted for about 40% and 70% of the maximum cycling power of older adults, which were common levels for daily aerobic exercises [26,27]. These three cadences were also among the values commonly presented in daily cycling [27,28]. A break of at least 5 min was allotted between each trial, and the subject’s fatigue effect was monitored by using a heart rate monitor (Magene, Magene H64, Tsingtao, Shenzhen, China). The subject would not continue the test until they recovered to the resting heart rate. If necessary, a longer break time was permitted.

### 2.3. Data Collection

Electromyography (EMG) signals, functioning as sensors, capture muscle electrical activity through electrodes, providing real-time insights into muscle activity levels and functional states. These signals are crucial for movement control, fatigue assessment, and rehabilitation monitoring. EMG signals enable dynamic tracking of muscle conditions and offer analytical insights into muscle coordination and performance, which are essential for optimizing exercise training and rehabilitation programs. Additionally, in clinical settings, EMG signals are used for diagnosing muscle disorders and neurological issues, offering critical evaluative data. Thus, EMG signals, as sensors, hold significant academic and practical value in both sports science and medical fields.

**Figure 2:**
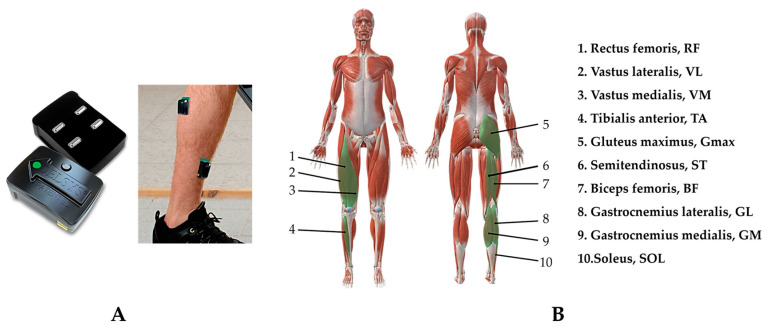
(**A**) Delsys EMG system; (**B**) overview of EMG data collection.

A wireless EMG system (Delsys Trigno Wireless System, DELSYS, Boston, MA, USA) (Figure 2A) was used to obtain surface EMG signals of 10 muscles in the dominant lower limb at 2000 Hz. Sensors were installed only on the dominant side of the subjects, and data collection was limited to this side. Dominant lower limb refers to the leg that exhibits higher functionality and coordination during activities, typically the leg that is used more frequently in standing, walking, or performing movements. The EMG system consists of wireless Ag electrodes arranged in parallel bar (contact area: 1 × 10 mm; distance between electrodes: 10 mm). Subjects underwent skin preparation, including shaving and cleaning with alcohol. Electrodes were then placed over the muscle belly aligned with the muscle fiber orientation according to SENIAM recommendations [29], attached to the skin with double-coated tape, and fixed with athletic tape. The muscles recorded included the gluteus maximus (Gmax), semitendinosus (ST), biceps femoris (BF), vastus medialis (VM), rectus femoris (RF), vastus lateralis (VL), gastrocnemius medialis (GM), gastrocnemius lateralis (GL), soleus (SOL), and tibialis anterior (TA) (Figure 2B). One retroreflective marker (14 mm) was placed on each end of the lateral center of the pedals to measure the crank angle. The markers’ trajectory was measured at 200 Hz by a Vicon motion capture system (Vicon Motion Analysis, Oxford, UK). The Vicon system and Delsys system were synchronized. In each trial, the data were recorded for 30 s once the steady cadence was reached.

We collected EMG and kinematic data from 32 participants under six different cycling tasks. To ensure the reliability and representativeness of the results, each cycling task was recorded 2–3 times, with each recording lasting 30 s. The kinematic data are stored in the QTM file format, while the EMG data are stored in MATLAB and Excel formats. We plan to organize the data and make them available for interested researchers in the future.

### 2.4. Data Preprocessing and Muscle Synergy Extraction

Visual3D (C-Motion, Inc., Germantown, MD, USA) was used to calculate kinematic variables. A second-order Butterworth low-pass filter with a cutoff frequency of 6 Hz was used to preprocess the 3D marker coordinates and segment the cycling cycles from the dataset. Here, a complete pedal cycle is described as the rotation process starting from the top dead center (the highest vertical position of the crank), passing through the bottom dead center (the lowest vertical position of the crank), and returning to the top dead center. The EMG data were extracted for 10 consecutive pedal cycles.

EMG data were preprocessed using a custom script in Matlab (R2019b, MathWorks, Natick, MA, USA). Initially, the EMG signals underwent band-pass filtering (10–400 Hz) to eliminate low-frequency and high-frequency noise, ensuring signal quality. This was followed by full-wave rectification to convert the signals to non-negative values, and root mean square (RMS) calculation to produce a linear envelope representing the overall muscle activity level. Subsequently, to standardize the EMG data across different experimental conditions, the data from each muscle were normalized by its maximum peak value observed across all pedaling tasks [30]. Additionally, the time scale was normalized by interpolating 101 points within each cycle to ensure consistency and comparability of the data across the time dimension [31,32].

A non-negative matrix factorization (NNMF) algorithm was employed to identify muscle synergy from the preprocessed EMG matrix [33]. NNMF is regarded as the most appropriate method because it best captures the variations in muscle activity with speed and reconstructs the muscle activation patterns [34]. This algorithm decomposes the multidimensional EMG matrix into a linear combination of k muscle synergy vectors (W) and their corresponding synergy activation coefficients’ (H) curves. The final EMG signal collected from each single-channel surface EMG is a superposition of these k linear combinations. This equation was as follows (1):

(1) $$E_{m\times n}=W_{m\times k}\times H_{k\times n}+e$$

where E is an m × n EMG matrix representing the activation patterns of m muscles at n time points; W denotes the relative activation weights of m muscles within each of k muscle synergies (k is the number of synergies); H represents the activation curves corresponding to each W, illustrating the relative contribution of each synergy for the overall muscle activity pattern; and e is the residual error matrix. W was normalized by its maximal value to ensure that all the values in the W were between 0 and 1. In this study, there are 10 channels of EMG signals, so the value of k is taken as an integer between 1 and 10, and the original matrix E is subjected to non-negative matrix factorization 25 times for each value of k. When the e between the reconstructed matrix E’ and the original matrix E is iterated to the minimum, the current W and H are the final results under the current number of k.

Variance accounted for (VAF) is commonly used to assess the degree of reconstruction of the matrix E’ relative to the matrix E, where E is the original matrix representing W × H + e, and E’ is the reconstruction matrix representing W × H. We varied the number of synergies, k, from 1 to 10 and calculated the VAF values. The k was selected as soon as the overall VAF exceeded 85% and the muscle VAF (VAFmuscle) exceeded 75% [24,35]. At this point, the chosen k was considered the optimal number of synergies for the subject under the current conditions. VAF was determined as follows (2):

(2) $$VAF=1-\frac{\sum_{i=1}^{m}\sum_{j=1}^{t}{\left(e_{i,j}\right)}^{2}}{\sum_{i=1}^{m}\sum_{j=1}^{t}{\left(E_{i,j}\right)}^{2}}.$$

Then, we matched the synergies of all subjects in each group one by one by Pearson’s correlation coefficient to obtain k groups of synergies [36]. Finally, the activation order of the k synergies was determined by the activation time of H, forming a schematic diagram of the k muscle synergies corresponding to the cycling cycle.

### 2.5. Assessment of Inter-Individual and Intra-Individual Variability

Inter-individual variability refers to the differences observed among multiple participants when performing the same movement, and intra-individual variability pertains to the variations in movement patterns observed within the same participant across multiple repetitions of the same task [37]. To quantify the variability of W and H, we calculated the variance ratio (VR). VR is not influenced by peak amplitude and effectively assesses the overall waveform repeatability, making it an appropriate index for analyzing inter-individual and intra-individual variability [38,39]. The range of VR values is 0–1, with a lower VR value reflecting lower variability. VR was determined as follows (3):

(3) VR=∑i=1k∑j=1sXij−X¯i2k(s−1)∑i=1k∑j=1sXij−X¯2(ks−1) with X¯=1k∑i=1kX¯i

Inter-individual variability was computed separately for each cycling condition, where k represents the number of measurements (i.e., 10 for W and 100 for H), and s denotes the number of subjects (i.e., 16). $\bar{X}$_i_ denotes the average value of the ith sample across 16 subjects, and X_ij_ denotes the value of the ith sample for the jth subject. Intra-individual variability was calculated separately for each subject, where s represents the number of tasks (i.e., 6). $\bar{X}$_i_ denotes the average value of the ith sample across six tasks, and X_ij_ denotes the value of the ith sample in the jth task.

### 2.6. Statistical Analysis

The Mann–Whitney test was applied to explore potential differences in intra-individual variability of W and H between groups. Depending on whether the data follow a normal distribution, either an ANOVA or a Kruskal–Wallis H test was used to compare W across age groups. The intergroup differences in H were compared using statistical parametric mapping. An alpha level of 0.05 was considered statistically significant.

## 3. Results

### 3.1. The Extraction of Muscle Synergies

**Figure 3:**
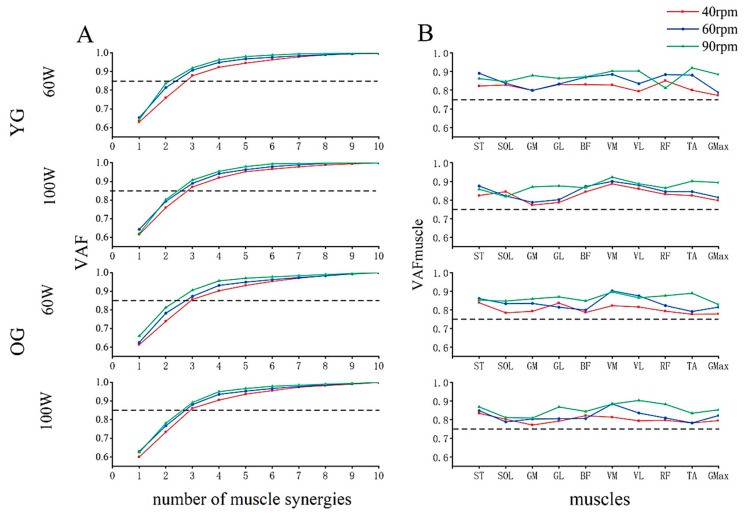
Overall VAF for different numbers of synergies (**A**) and VAF_muscle_ for three muscle synergies (**B**). YG: young group; OG: older group; VAF: variance accounted for; ST: semitendinosus; SOL: soleus; GM: gastrocnemius medialis; GL: gastrocnemius lateralis; BF: biceps femoris; VM: vastus medialis; VL: vastus lateralis; RF: rectus femoris; TA: tibialis anterior; Gmax: gluteus maximus.

In Figure 3A, we can find that when the number of synergies is three, the VAF values for all riding conditions were above the horizontal line of 0.85 for both populations, and three synergies led to VAF_muscle_ ranging from 0.771 ± 0.034 (GM during the 40 rpm at 100 W on OG) to 0.923 ± 0.046 (VM during the 90 rpm at 100 W on YG) (Figure 3B). Therefore, we extracted three synergies for young and older adults. 

**Figure 4:**
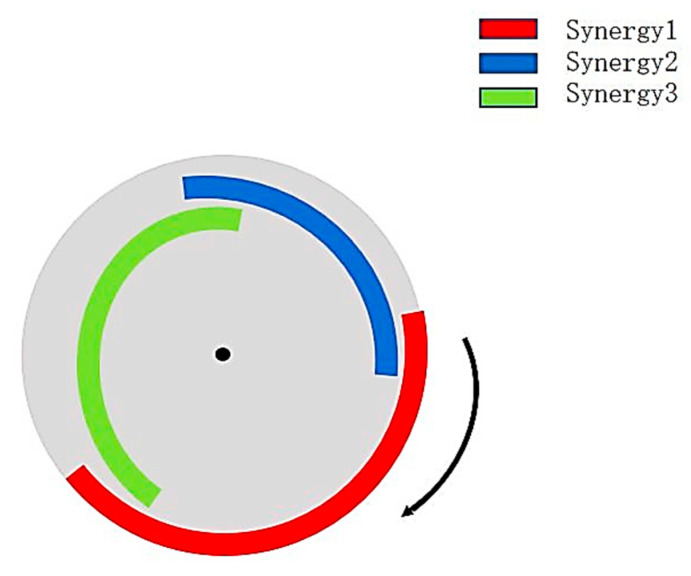
Schematic of the three muscle synergies corresponding to cycling cycles.

**Figure 5:**
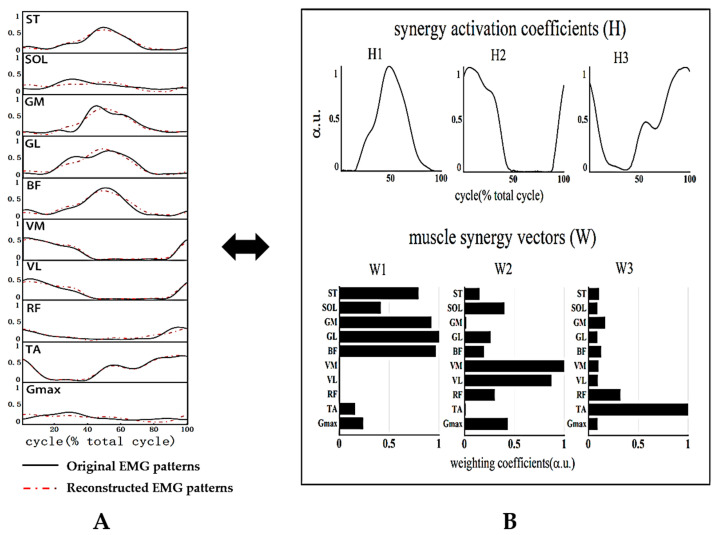
An example of muscle synergy extraction. (**A**) Original and reconstructed EMG curves. (**B**) Extracted muscle synergies. ST: semitendinosus; SOL: soleus; GM: gastrocnemius medialis; GL: gastrocnemius lateralis; BF: biceps femoris; VM: vastus medialis; VL: vastus lateralis; RF: rectus femoris; TA: tibialis anterior; Gmax: gluteus maximus; W1, W2, and W3 represent synergy vectors one, two, and three, respectively; H1, H2, and H3 represent activation coefficients one, two, and three, respectively.

Muscle synergies were activated in the order of 2–1–3 with the pedaling cycle (Figure 4). The original and reconstructed EMG curves and extracted muscle synergies are shown in Figure 5. The extracted muscle synergies were named W1, W2, W3 and H1, H2, H3, respectively.

### 3.2. The Inter-Individual Variability Comparison in Muscle Synergy

In W, older adults had lower inter-individual variability in synergy two than the younger adults, and the inter-individual variability of synergy two was higher when cycling at the power of 60 W than 100 W in all groups. Considering the inter-individual variability of H, we found that older adults had higher variability in synergy three than the younger adults under most cycling conditions except during the 90 rpm at 60 W cycling task (Table 2).

### 3.3. The Intra-Individual Variability Comparison in Muscle Synergy

**Figure 6:**
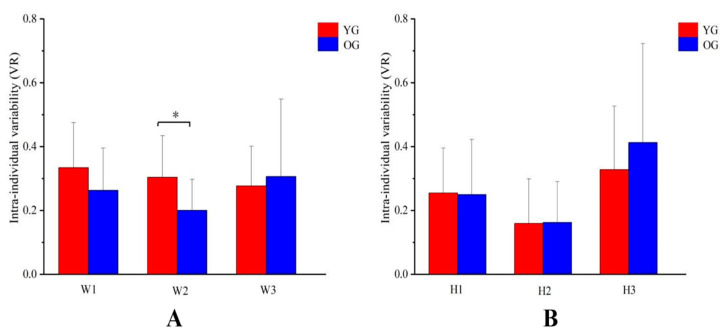
Intra-individual variability (VR value) of W (**A**) and H (**B**) for the young and older groups. W1, W2, and W3 represent synergy vectors one, two, and three, respectively; H1, H2, and H3 represent activation coefficients one, two, and three, respectively; YG: young group; OG: older group; W: muscle synergy vectors; H: synergy activation coefficients. * indicates a significant difference.

The intra-individual variability of W2 was significantly lower in older adults (*p* = 0.032), and H3 in older adults showed a trend towards higher variability (Figure 6).

### 3.4. Analysis of Muscle Synergy Structures

**Figure 7:**
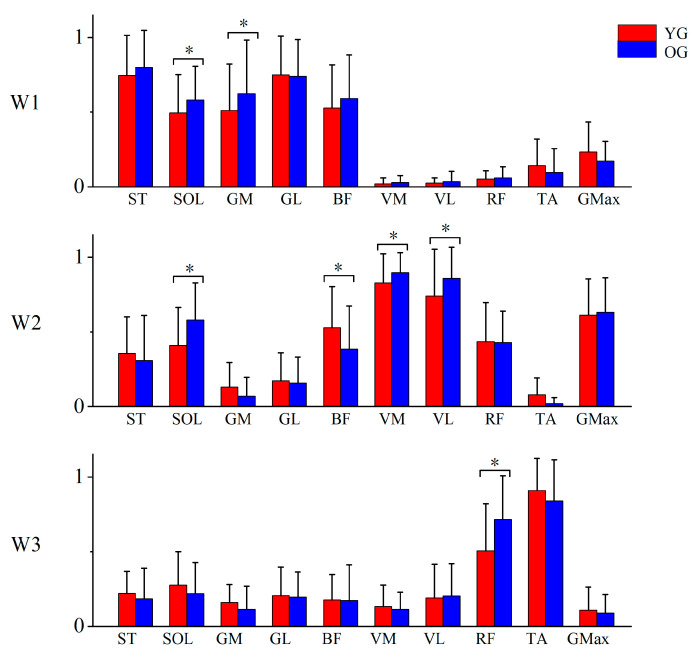
Comparison of W in the young and older groups; W1, W2, and W3 represent synergy vectors one, two, and three, respectively; YG: young group; OG: older group; ST: semitendinosus; SOL: soleus; GM: gastrocnemius medialis; GL: gastrocnemius lateralis; BF: biceps femoris; VM: vastus medialis; VL: vastus lateralis; RF: rectus femoris; TA: tibialis anterior; Gmax: gluteus maximus. * indicates a significant difference.

W2 mainly involved the activity of three knee extensors (VM, VL, and RF) and one hip extensor (Gmax), along with minor activation from SOL, which contributed to the propulsion during the downstroke phase. W1 mainly utilized three ankle flexors (SOL, GM, and GF) and two knee flexors (i.e., ST and BF), which were active in the latter part of the downstroke stage and the extension-to-flexion transition. W3 primarily included RF and TA, which were active during the upstroke phase and the transition phase to cycle end (Figure 7). Comparing the weighting values of dominant muscles in each W, we discovered that the weighting of SOL (*p* = 0.027) and GM (*p* = 0.021) in W1; SOL (*p* < 0.001), VM (*p* = 0.010), and VL (*p* = 0.007) in W2; and RF (*p* < 0.001) in W3 was significantly higher in the older group than in the younger group, while the weighting of BF (*p* = 0.002) in W2 was lower in the older group (Figure 7). 

**Figure 8:**
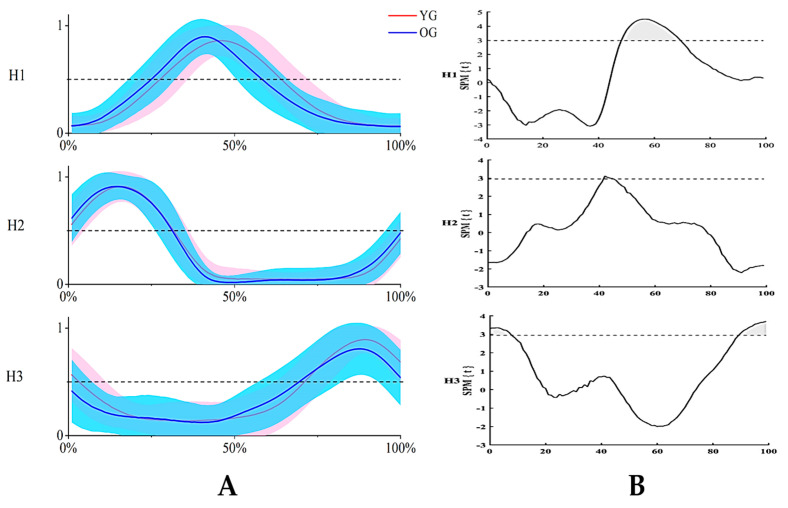
(**A**) Comparison of H in the young and older groups. (**B**) Statistical differences in H calculated using spm1d; the gray shaded area represents *p* < 0.05, a = 0.05. H1, H2, and H3 represent activation coefficients one, two, and three, respectively; YG: young group; OG: older group.

In Figure 8B, the dashed line represents the t-value corresponding to *p* = 0.05, and the gray shaded area represents *p* < 0.05. We found that there were notable differences between the young and older groups from 48% to 69% of H1 and from 0% to 8% and 89% to 100% of H3 (*p* < 0.05). To be specific, compared with the younger group, older adults showed a forward-shifting peak in H1 and a lower peak magnitude in H3 (Figure 8A).

## 4. Discussion

Our study investigated the number, variability, and structure of lower-limb muscle synergy in young and older adults during various cycling conditions. The results indicated that aging did not influence the number of synergies. In W, we observed significantly lower variability in synergy two among older adults than younger adults. In H, the older adults only exhibited a tendency towards higher variability in synergy three, partially aligning with our hypothesis. Further analyses of muscle synergy structure showed increased weighting in major contribution muscles, the forward-shifting peak of H1, and lower peak magnitude in H3 among older adults.

Three synergies were extracted from young and older adults during cycling (Figure 3), which was consistent with previous studies for young adults [40,41]. Esmaeili et al. [42] and Zych et al. [31] identified four or five muscle synergies during cycling tasks, which may be attributed to their collection of data from both sides of the lower limbs, whereas we only collected data from one side of the lower limbs.

The muscle synergy variability and structure could serve as a more sensitive measure, capturing subtle alterations in neuromuscular control strategies before alterations in the number of synergies. W, representing the relative weight of each muscle in each synergy, was less variable among older adults than younger adults for W2 (Table 2, Figure 6). Then, we also observed that the older adults showed increased activation in agonistic muscles—VM, VL, and SOL—and reduced activation in antagonist muscles—BF—in this synergy (Figure 7). 

We interpreted these differences as a compensatory strategy developed by older adults to cope with cycling resistance, due to the fact that W2 is the main synergy in generating energy to propel the crank [43], in which a group of muscles comprising three knee extensors (VM, VL, and RF), one hip extensor (Gmax), and one ankle plantar flexor (SOL) was synergistically activated. Less variability and greater activation in W2 might mean that more older adults need to make full use of W2, exerting the function of VM, VL, and SOL as monoarticular muscles to extend the knee joint and to plantarflex the ankle joint, to facilitate the propulsion phase. Young adults, however, did not require as much activation of the synergy or these monoarticular muscles to generate the same power, so these muscles could be used for other functions according to individual’s demand, resulting in higher inter- and intra-individual variability in young adults. These results corresponded to a prior study that analyzed muscle synergy during step ascent [24], showing higher intra-individual similarity across step heights in older adults. However, Guo et al. [18] did not find more associated synergies like W2 during walking in older adults, possibly because walking posed a greater challenge in maintaining balance, rather than demanding the same level of muscle strength as resistance cycling or climbing. 

In this study, we also discovered that when cycling at the power of 60 W, there was higher inter-individual variability in W2 for young and older adults than when cycling at 100 W (Table 2). This was consistent with the result from Turpin’s study [44] that the alteration in power output was linked to greater similarities in W. When workloads increase, greater muscle activation and more consistent synergy in the propulsive phase were demanded to generate more power. 

H represents the relative contribution of each synergy for the overall muscle activity pattern. Higher variability in H might be associated with increased functional demands on older adults’ ability to adapt to decreased physical capacity and challenging cycling conditions. Additionally, older adults had lived longer than the younger ones, accumulating various motor control adjustment patterns. Their different sensorimotor histories or varying degrees of degenerative changes might have altered neuromuscular control, as noted by Guo et al. [18]. In our study, synergy three, comprising RF and TA and responsible for the upstroke and upstroke/downstroke transition phases, showed higher variability, which was in accordance with the higher variability of effective force acting on the pedal during these two phases [45]. Compared with the younger group, there was a trend towards higher inter- and intra-individual variability and decreased peak magnitude in H3 in older adults (Table 2, Figure 6). This might be due to synergy three being in the opposite position of synergy two in the cycle, where older adults did not carry out the pull-up action due to the intervention of the other leg as a power producer, with lower variability and greater muscle activation in W2 as described above, which might partly reflect the poorer coordination of two lower limbs in older adults [43].

Synergy one, grouping three ankle flexors (SOL, GM, and GF) and two knee flexors (i.e., ST and BF) in a module, was activated from the latter part of the downstroke phase following synergy two and reached the peak activation in the downstroke/upstroke transition phase [43]. We found that the peak activation of W1 occurred earlier (Figure 8), and the activation amplitudes of SOL and GM in W1 were larger (Figure 7) in older adults than in younger adults. These indicated that older people called more ankle plantarflexion functions in synergy one during the latter part of the propulsion phase, which could be considered a compensatory strategy for synergy two to propel the crank.

This study has some limitations. First, the sample was small and specific, limiting the extrapolation of the results to the general population. Future research could expand the sample and include a broader range of age and weight categories for more comprehensive analysis. In addition, the EMGs were measured only from the unilateral lower limb. Although cycling was typically considered a symmetrical task in healthy adults, asymmetrical manifestations in symmetrical movements may be exacerbated with age, especially during challenging activities. Lastly, our study did not investigate the impact of covariates such as muscle strength, endurance, cardiorespiratory fitness, etc., on muscle synergy. Therefore, we should be cautious about attributing all these results to aging. We also suggest that further studies could introduce EEG or fNIRS into muscle synergy analysis to explore functional brain activation during the propulsion stage of cycling in the elderly.

## 5. Conclusions

To produce the same cycling power and cadence as younger individuals, older adults make adaptive adjustments in muscle control, including increased weighting in major contribution muscles in all synergies, greater consistency in the use of primary force-producing synergies, and earlier peak activation of subsequent synergy, which appeared to compensate for diminished efficiency in muscle force production during the propulsion phase of cycling in older adults.

This finding enhances our understanding of the mechanism of muscle strength decline in the elderly and provides a theoretical basis for the prevention and treatment of muscle degeneration in the elderly. Given the limitations of the sample, future studies with more heterogeneous populations are recommended to give more general validity to our conclusions. A more comprehensive understanding of the changes in muscle synergy patterns during cycling in older adults in the general population can aid in providing guidance for training designing, even exercise rehabilitation.

## Figures and Tables

**Table 1:** Basic information of two groups.

	$\mathrm{Age}\;(\mathrm{Years})\;(\bar{X}\;$± SD)	$\mathrm{Weight}\;(\mathrm{kg})\;(\bar{X}\;$± SD)	$\mathrm{Height}\;(\mathrm{cm})\;(\bar{X}\;$± SD)
YG	22.8 ± 1.1	65.3 ± 2.5	173.5 ± 1.9
OG	63.3 ± 1.5	63.4 ± 2.2	171.2 ± 2.1

YG: young group; OG: older group.

**Table 2:** Inter-individual variability (VR value) of W and H in all cycling conditions for the young and older groups.

**A: Inter-individual variability of W and H at 60 W**									
	40 rpm			60 rpm			90 rpm		
	#1	#2	#3	#1	#2	#3	#1	#2	#3
W									
YG	0.278	0.535	0.409	0.363	0.514	0.330	0.263	0.621	0.277
OG	0.435	0.394	0.362	0.367	0.370	0.506	0.252	0.367	0.349
H									
YG	0.340	0.186	0.197	0.178	0.373	0.233	0.200	0.075	0.316
OG	0.485	0.120	0.286	0.228	0.095	0.417	0.090	0.066	0.238
**B: Inter-individual variability of W and H at 100 W**									
	40 rpm			60 rpm			90 rpm		
	#1	#2	#3	#1	#2	#3	#1	#2	#3
W									
YG	0.371	0.391	0.260	0.367	0.449	0.435	0.366	0.371	0.413
OG	0.258	0.221	0.240	0.257	0.270	0.433	0.192	0.334	0.500
H									
YG	0.402	0.086	0.143	0.254	0.059	0.330	0.255	0.052	0.536
OG	0.256	0.047	0.250	0.211	0.068	0.551	0.315	0.245	0.655

YG: young group; OG: older group; W: muscle synergy vectors; H: synergy activation coefficients; #1, #2, and #3 represent synergy one, two, and three, respectively.

## Data Availability

The data that support the findings of this study are available on request from the corresponding author.
